# A randomized prospective study of extended tocopherol and pentoxifylline therapy, in addition to carbogen, in the treatment of radiation late effects

**DOI:** 10.3332/eCMS.2008.81

**Published:** 2008-06-09

**Authors:** S Brennan, O Salib, C O’Shea, M Moriarty

**Affiliations:** St Luke’s Hospital, Dublin, Ireland

## Abstract

**Purpose:**

pentoxifylline (PTX) and tocopherol (vitamin E) are antioxidants previously shown to be useful in combination in the treatment of late radiation induced toxicity. The purpose of this study was to investigate the benefit of combination therapy with carbogen pentoxifylline and tocopherol in the mitigation of late radiation effects. As the optimal duration of PTX and tocopherol treatment has not been fully established, we studied short versus extended treatment duration.

**Methods:**

we conducted a phase II prospective randomized study of short versus prolonged treatment with pentoxifylline (800 mg) and tocopherol (1000 IU) orally once daily in patients with grade three toxicity post-radical radiotherapy. In addition, all 18 patients received inhaled carbogen (95% O + 5% CO_2_) over 90 minutes, five days/week, for three weeks. The primary end point was improved in maximum Lent-Soma toxicity scores.

**Results:**

maximum Lent-Soma scores improved in six of the 18 patients (response rate 33%). The proportion of patients responding to treatment in the prolonged treatment arm B was more than double than in the shorter arm A, but this did not reach statistical significance (p=0.321). Two patients who had prolonged treatment (arm B) had complete resolution of their symptoms, which was maintained at two and three year follow-ups.

**Conclusions:**

we recommend prolonged treatment for 12 months, with PTX and tocopherol in combination with carbogen therapy, in the management of late radiation effects.

## Introduction

As cancer treatment outcomes improve, there is increased emphasis on reducing toxicity of treatment as part of an effort to broaden the therapeutic index of therapy. Late radiotherapy effects are a considerable source of symptomatic morbidity in survivors of cancer therapy. Although traditionally considered progressive and irreversible, there is now mounting evidence to support the potential reversibility and treatment of radiotherapeutic injury using antioxidant therapy [1,2].

Over the past decade, there have been several reports in support of tocopherol and pentoxifylline (PTX) in the treatment of late radiation effects [1,2,3]. Hyperbaric oxygen has also shown some efficacy in this area [4]. Carbogen is thought to act similarly to hyperbaric oxygen (HBO) by reversing tissue hypoxia and thus removing free radicals, which are implicated in the pathogenesis of late radiation effects [5].

The combination of PTX and tocopherol has yielded far greater and consistent results than the use of either agent alone [1]. We postulated that the addition of carbogen therapy to this combination would further enhance the modulation of radiation-induced normal tissue damage. The optimum duration of treatment has not been fully established, with trials continuing treatment for a variety of times from three to 36 months [2].

We conducted a phase II prospective randomized study, examining the benefit of extended compared to short-term use of PTX and tocopherol used in combination with carbogen therapy, in the management of radiation-induced normal tissue morbidity.

## Methods

The study design involved a prospective, randomized trial of short versus prolonged treatment, with pentoxifylline and tocopherol in addition to inhaled carbogen (95% O + 5% CO_2_). Carbogen was inhaled via a close-fitting face mask, with a one-way valve and closed breathing system over 90 minutes/day, five days/week, for three weeks. Patients were randomized to either arm A, which consisted of a short three-week course of pentoxifylline and tocopherol, or arm B, which was a prolonged 12-month course. Pentoxifylline was administered at a dose of 800 mg and tocopherol at a dose of 1000 IU. Both were taken orally once daily, and the same dose was used in both arms of the study.

**Figure 1:**
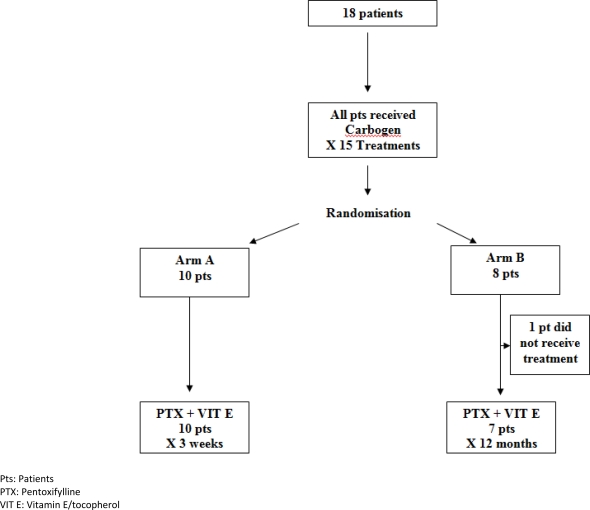
The randomization process

Patients with grade 3 (Lent-Soma) [6] toxicity, post-radical radiotherapy, for a variety of cancer primaries, were eligible for the trial. Inclusion criteria required that patients had received radical radiotherapy that consequently caused grade 3 side effects within the irradiated area. Patients were accrued as a consecutive presenting sample. Eighteen patients with significant late morbidity post-radical radiation therapy were randomized to one of the two study arms, using envelope randomization (see Figure 1).

Figure 1 depicts the randomization process. All 18 patients in the study received carbogen therapy (95% O + 5% CO_2_) over 90 minutes, five days/week, for three weeks, which was inhaled via a close-fitting face mask, with a one-way valve and closed breathing system, over 90 minutes daily five days/week, for three weeks. Patients were randomized to either arm A, which consisted of a short three-week course of pentoxifylline and tocopherol, or arm B, which was a prolonged 12-month course. Pentoxifylline was administered at a dose of 800 mg and tocopherol at a dose of 1000 IU. Both were taken orally once daily, and the same dose was used in both arms of the study.

We initially planned to include 30 patients in the trial. However, due to slow accrual the study was closed after 18 patients. One of the reasons for slow accrual was patients’ reluctance to attend the hospital day ward 15 times over a three-week period for carbogen therapy. The study commenced in March 2001 and randomization completed in August 2003. The mean time period between completion of radiotherapy and study enrolment was 58.6 months (range 11–224 months). Of the 18 patients, three were male and 15 were female. The mean age was 59.9 years (range 22–82 years). The group included eight patients with breast cancer, four patients with head and neck cancer, four patients with gynaecological malignancies, one patient with gastro-intestinal malignancy and one case of Bowen’s disease of the skin. Radiation-induced toxicities included subcutaneous fibrosis lymphoedema, post-radiation cystitis and increased bowel frequency (see Table 1).

The severity of radiation late effects was assessed using the Lent-Soma (late effect normal tissues subjective objective management and analytic) scale, defined by the Radiation Therapy Oncology Group and the European Organisation for the Research and Treatment of Cancer [6]. Clinical assessment of late side effects, recorded using Lent-Soma scales, was used as the primary end point. Maximum Lent-Soma scores were recorded at baseline and at four, eight and 12 months post-treatment. One patient did not receive any treatment as she was hospitalized for treatment of an infection. Intention to treat analysis was performed. Patient’s data were entered onto EXCEL and transferred to Minitab statistical software for statistical analysis. We compared the overall proportions of responders in each arm of the trial, using Fisher’s exact test with a significance level of p=0.05.

Written information consent was obtained from all patients. Ethical approval was obtained from St Lukes Ethics and Medical Research Committee.

## Results

All patients tolerated carbogen therapy extremely well. Three patients experienced nausea due to pentoxifylline, one of whom discontinued treatment. The other two patients improved on commencing domperidone medication. We subsequently adopted a policy of administering five days of domperidone to all patients routinely at the start of therapy. Following this there were no further reports of nausea.

Of the 17 patients who received treatment, six patients had improvement in their symptoms (see Tables 1 and 2), thus giving an overall response rate of 33%. The overall proportions responding in each arm were 2/10 in arm A versus 4/8 in arm B. Although the proportion responding to treatment in the prolonged treatment arm B was more than double (0.5 versus 0.2) than in the shorter arm A, (see Table 3) the difference is not statistically significant (p=0.321).

The median time to onset of response was 3.5 months with a range of 2–7 months (see Table 1). Of the two patients in the shorter treatment group A arm who responded, the first (patient 5) had dysuria and diarrhoea, which showed an initial improvement from grade 3 to grade 2 after two months, but her symptoms subsequently recurred. She was later re-treated with 15 sessions of carbogen over a three-week period, and 12 months of pentoxifylline (800 mg) and tocopherol (1000 IU). On re-treatment, with the extended regimen, her dysuria reduced to grade 1 and this benefit was maintained for over one year. The second patient (patient 14) had fibrosis post-breast radiotherapy, which improved from grade 3 to grade 2 toxicity at two months, but then subsequently refused further follow-up for personal reasons.

Four of the eight patients in the prolonged 12-month treatment arm B demonstrated response to treatment. Two of these patients with grade 3 fibrosis (patients 17 and 18 in Table 1) had complete resolution of their symptoms, and this benefit was maintained for two and three years, respectively. The third patient (patient 6) had urinary incontinence, which improved from grade 3 to grade 2 toxicity, and this was maintained for one year. The final patient (patient 4) had dysuria and PV bleeding, which improved from grade 3 to grade 2, but this benefit was not maintained.

## Discussion

Late radiation effects are progressive in nature and have a significant negative impact on quality of life in long-term cancer survivors. In our study, 33% of patients had an improvement in their symptoms, which otherwise would have remained unchanged or perhaps even worsened. We feel that the combination of carbogen with PTX and tocopherol represents an effective treatment, especially as treatment was well tolerated, cost-effective and no established alternative currently exists. Although nausea was a side effect of the PTX treatment, this responded well to treatment with domperidone.

Late radiotherapy effects are a debilitating, chronic, adverse effect of treatment and have traditionally been considered both progressive and irreversible. In such circumstances, prevention is often the best cure and limiting both dose and volume to irradiated tissues is imperative in reducing the incidence and extent of such effects. Despite these efforts, there is much individual variation and unpredictability in regard to development of late radiotherapy side effects. Treatment intensification is becoming increasingly popular both in terms of concurrent chemotherapy and intensified fractionation schemes. However, this will likely be at the expense of increased incidence and severity of late radiotherapeutic injury.

The irreversibility of late radiation effects has been disputed for some time [1,2]. Radiation induced injury is traditionally thought to be a chronic progressive process due to mitotic cell death in tissues and reduction in the number of surviving clonogens. However, in recent times, there is increasing evidence to suggest that this process is not entirely irreversible [3], and that late radiation effects are due to a much more dynamic interactive process between multiple cell types, including parenchymal and vascular cells. This is enhanced by secretion of a cascade of pro-inflammatory and fibrogenic cytokines [7].

As an anticytokine pentoxifylline is thought to reverse this process [8]. PTX is a haemorheologic methylxanthine derivative initially developed to improve blood viscosity and microcirculatory disorders. Alpha tocopherol is a powerful antioxidant that protects membrane phospholipids from oxidative damage [9] and acts as a scavenger of reactive oxygen species. The precise mechanism of action of these agents is not yet known. Both PTX and tocopherol are antioxidants previously shown to be of superior benefit in combination in the treatment of late radiation effects [3]. In a clinical trial, Delanian *et al* reported objective response rates of 53% at six months, using this combination [10], that is regression of radiation-induced fibrosis surface area. Our study reports a response rate of 33%, giving further confirmation of the efficacy of this combination in the treatment of late radiotherapy effects.

Hyperbaric oxygen therapy (HBOT) has been used to treat several medical conditions, including necrotising fasciitis and osteoradionecrosis [11]. It has been used in radiation oncology, as both a radiosensitiser and also as a treatment of late radiation effects. It is thought to stimulate angiogenesis and improve tissue oxygenation, resulting in healing of damaged soft tissue bone and cartilage. In a small placebo controlled prospective trial, Carl *et al* reported significant improvements in pain and swelling in 44 patients with late radiation induced injury post-radiotherapy for breast cancer [4]. In another retrospective trial of 75 patients with osteoradionecrosis, 81% of patients responded to HBOT [12]. The Consensus Conference, led by the European Society for Therapeutic Radiology and Oncology and the European Committee for Hyperbaric Medicine, recommended prospective collection of toxicity data and setting up of multi-centre randomized controlled trials to obtain higher levels of evidence for indications of HBOT [11].

Carbogen is thought to work in a similar manner to HBOT. In a study by Collet *et al*, there was no difference in results between hyperbaric oxygen and slightly pressurized air, and the two treatments were considered equally effective [13]. Powel *et al* conducted a study that showed breathing carbogen (5% CO_2_ and 95% O) increased tumour oxygenation, by comparing pO_2_ measurements pre- and post-carbogen breathing [5]. In our study, carbogen therapy was tolerated well. As patients were not randomized for carbogen therapy, it is not possible to assess the relative contribution of carbogen to the results obtained.

The optimum duration of treatment has not been fully established, with other trials continuing treatment for various durations from three to 36 months [2]. This trial showed a trend towards better response with more prolonged treatment, with three of the four patients in the prolonged treatment arm B, maintaining their improvements for 1–3 years. Also, the proportion responding to treatment in the prolonged treatment arm B was more than double that in the shorter arm A. This result was not statistically significant, possibly due to the small number of patients in the study and also the combination of patients with a variety of late toxicities in this study. However, this finding is consistent with other studies [2] and gives further confirmation of the benefit of prolonged treatment. We recommend clinical use of these agents with treatment duration of at least 12 months.

In addition, there is evidence for rebound effects in this study if the treatment period is too short. One patient had a recurrence of her symptoms on completion of the shorter three-week course, suggesting a rebound effect. In a study of response kinetics in long-term treatment with PTX and tocopherol, Delanian *et al* also reported a rebound effect due to discontinuation of treatment in three of seven patients in the shorter treatment arm [2]. The same patient in our trial had an even more substantial improvement from grade 3 to grade 1 toxicity (diarrhoea) on re-treatment with this therapy for 12 months, thus giving further basis for more prolonged therapy.

This was a heterogeneous group of patients indicating the broad scope of potential benefit with this treatment. Other groups have reported improvement in 71% of patients with proctitis and enteritis with combination PTX and tocopherol therapy [14]. Gothard *et al* have shown significant improvements in Lent-Soma scores for genito-urinary dysfunction and proctitis with this combination [15]. This combination has also been studied in osteoradionecrosis [16], and pentoxifylline has been shown to be of benefit in radiation-induced trismus [17] and lung toxicity in patients with breast and lung cancer [18].

However, several studies have examined the use of PTX and tocopherol specifically in the management of radiation-induced subcutaneous fibrosis [1,2 and 11]. We would support this as, in our study, two patients with radiation-induced fibrosis both had complete resolution of their symptoms on the prolonged treatment arm B. These improvements were maintained for two and three years, respectively, and thus they represent the most significant responses in our trial. There has been much progress in understanding the molecular mechanisms and pathophysiology involved in radiation injury, and this should be used to model therapeutic strategies for this disease. In the era of targeted therapies, agents such as angiotensin-converting enzyme (ACE) inhibitors and angiotensin receptor type I antagonists, which target the renin-angiotensin system, are proving to be of benefit in modulating late radiation effects in kidney and lung tissues [19]. These tissues are known to possess an intrinsic renin-angiotensin system. ACE inhibitors are also being studied in radiation-induced lung injury in the RTOG 0123 study [20]. Thus, it may be possible to direct the use of different aetiology-based treatments to tissue-specific late radiation reactions. In this instance, we would suggest that combination therapy with carbogen PTX and tocopherol might be of greater benefit in the management of radiation-induced subcutaneous fibrosis. Future studies with this therapeutic combination should involve sub-group analysis for late radiation effects in different tissue types, as perhaps not all radiation-induced toxicity will respond in a similar manner.

## Figures and Tables

**Table 1:**
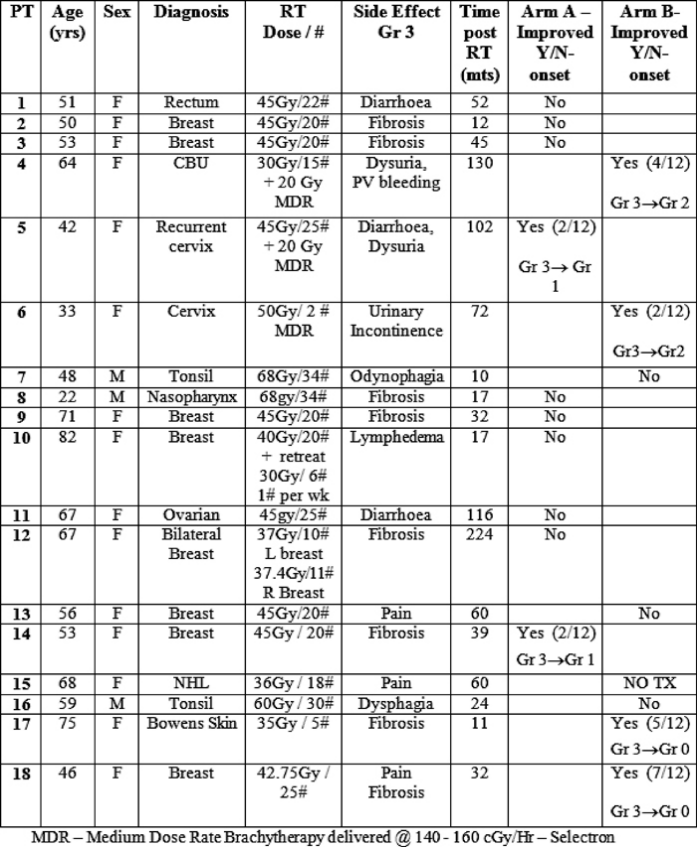
Overall results

**Table 2:**
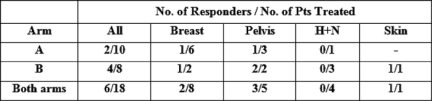
Proportion of patients responding to treatment according to diagnosis and treatment arm

**Table 3:**
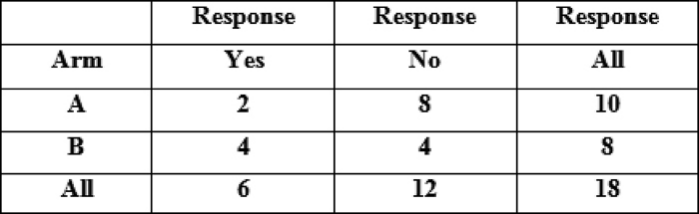
Results of Fisher’s exact test for comparison of proportions responding in each arm, using frequencies in frequency
